# How Do Violinists Adapt to Dynamic Assistive Support? A Study Focusing on Kinematics, Muscle Activity, and Musical Performance

**DOI:** 10.1177/00187208211033450

**Published:** 2021-08-04

**Authors:** Clara Ziane, Benjamin Michaud, Mickaël Begon, Fabien Dal Maso

**Affiliations:** 1 5622 Université de Montréal, Laval, QC, Canada; 2 Centre Interdisciplinaire de Recherche sur le Cerveau et l'Apprentissage (CIRCA), Montreal, QC, Canada; 3 Centre de Recherche du CHU Sainte-Justine, Montreal, QC, Canada

**Keywords:** motor control, biomechanics, electromyography (EMG), assistive technologies, upper extremity

## Abstract

**Objective:**

Assessing violinists’ motor and musical performance adaptations to dynamic assistive support (DAS) provided by a passive device, using a force-field adaptation paradigm.

**Background:**

Up to 93% of instrumentalists are affected by musculoskeletal injuries and particularly violinists. The repetitive nature of their work may lead to muscle fatigue, an injury risk factor. DAS has been used in occupational settings to minimize muscle activations and limit fatigue accumulation. DAS may however affect motor and musical performance.

**Method:**

Fifteen expert violinists were equipped with reflective markers and surface and intramuscular electromyography (EMG) sensors. Movements, muscle activations, and sound were recorded while participants completed three experimental conditions for which they continuously played a 13-s musical excerpt: *Control* (no DAS), *Adaptation* (DAS), and *Washout* (no DAS). DAS was applied at the left elbow (violin-holding side). Conditions were repeated 1 week later. Participants later listened to their own audio recordings playing with and without DAS and blindly assessed their performances. Linear mixed models were used to compare DAS and no-DAS conditions’ kinematic, EMG, and musical performance data.

**Results:**

DAS perturbed user kinematics but reduced mean activations of left medial deltoid and superior trapezius. Joint kinematic and muscle activation patterns between DAS and no DAS conditions however remained similar. Musical performance was unchanged with DAS.

**Conclusion:**

Though DAS modified violinists’ upper-limb configurations, resulting kinematics were not detrimental to musical performance. Reduced muscle activations with DAS could contribute to lessening muscle fatigue.

**Application:**

Although its effect on muscle fatigue should be further investigated, DAS might be useful in preventing violinists’ injuries.

## Introduction

Up to 93% of musicians are affected by playing-related musculoskeletal disorders (Kok et al., 2016). These can affect nerves, tendons, and muscles, causing physical pain while playing and sometimes threatening musicians’ careers (Mizrahi, 2020; Zaza, 1998). The amount of practice necessary to maintain the high-level performance of professional musicians (Kaufman-Cohen & Ratzon, 2011) inevitably leads to fatigue, one of the leading causes of musculoskeletal disorders (Côté, 2014). Violinists are particularly affected by these disorders (Ackermann & Adams, 2004; Zaza & Farewell, 1997), especially at the left shoulder and upper limb (Ranelli et al., 2011), as they combine multiple fatigue-inducing factors: quasi-static weight—the violin—holding, highly repetitive and often fast movements, and a constrained posture (Ackermann & Adams, 2004; Côté, 2014; Fry, 1986; Rensing et al., 2018). Risk factors being inherent to instrumental practice, adapting violinists’ working environments appears crucial to limit fatigue accumulation and, in turn, the prevalence of playing-related musculoskeletal disorders. Interestingly, dynamic assistive support (DAS) provided by mobility assistive devices, such as exoskeletons, has recently been implemented into the workplace to assist workers with drilling and load handling tasks (De Vries & De Looze, 2019). DAS successfully reduced muscle fatigue in two recent studies (Schmalz et al., 2019; Xiloyannis et al., 2019). It is thus likely that DAS would assist violinists in holding their instruments, possibly acting on fatigue-inducing factors such as static weight holding and posture constraints. Though promising, assistance provided by DAS may alter musicians’ motor outputs, which could be detrimental to musical performance and in turn make assistance unwarranted on the musical scene.

Only a few studies have assessed effects of DAS on both shoulder kinematics and muscle activations during dynamic upper-limb tasks such as load lifting, handling, and drilling (Hall & Crouch, 2020; Schmalz et al., 2019; Theurel et al., 2018). All found that DAS perturbs user kinematics but reduces agonist muscles’ activation levels when performing goal-oriented tasks (Hall & Crouch, 2020; Schmalz et al., 2019; Theurel et al., 2018). Indeed, Theurel et al. (2018) showed that load lifting and handling with an upper-body exoskeleton significantly changed the averaged elbow flexion-extension and shoulder adduction of seven workers whose jobs require box handling. Similarly, Schmalz et al. (2019) found that 12 novices showed increased shoulder abduction and elbow extension while drilling, and increased elbow extension while screwing nuts with a passive upper-body exoskeleton. It is possible that modifications to kinematics and muscle activity result from an early perturbation of the internal model, which is a goal-oriented sensorimotor representation of movement (Shadmehr & Mussa-Ivaldi, 1994), resulting in an increase in motor performance errors for tasks requiring precision (Scalona et al., 2018), such as drilling (Alabdulkarim et al., 2019). DAS may thus alter violinists’ joint kinematics and negatively impact their musical performance.

Repeated exposure to DAS could lead to progressive adaptation of violinists’ internal models, eventually enabling them to reach performance levels similar to those without DAS. Indeed, we know that participants performing a reaching task in the presence of externally imposed forces adapt to the new environment as observed by the gradual decrease in performance errors (Izawa & Shadmehr, 2011; Scalona et al., 2018; Shadmehr & Mussa-Ivaldi, 1994). In fact, repeated exposure to a force field allows participants to progressively update their internal models and by the end of an adaptation condition, their kinematics returns to baseline levels measured during a control null-field condition (Shadmehr & Mussa-Ivaldi, 1994). Additionally, Shadmehr and Mussa-Ivaldi (1994) showed that similarity scores, measured with correlation analyses, of reaching kinematics between a null-field control condition and a force-field adaptation condition went from moderate to strong throughout an adaptation period. Finally, the formation of a new internal model is confirmed by the sudden increase in performance errors during a washout condition, which is a null-field condition taking place right after the adaptation condition (Shadmehr & Mussa-Ivaldi, 1994). These aftereffects result from participants’ formations of new internal models during the adaptation period, which enabled them to perform the task in altered environmental conditions (Shadmehr & Mussa-Ivaldi, 1994). The internal model is however quickly readjusted to satisfy task demands of the washout condition (null field) and performance returns to initial baseline levels. Although washout also is an iterative process, it is faster than adaptation. Finally, studies have shown that participants adapted faster to a previously encountered perturbation when reexposed to it minutes, a day or even a week after initial adaption (Bastian, 2008; Huberdeau et al., 2015). This type of retention is called *savings*. Unfortunately, tasks performed in studies that have investigated motor adaptations do not accurately represent real-world tasks (Kitago et al., 2013; Shadmehr & Mussa-Ivaldi, 1994). Whether these results could generalize to violin playing, which requires tridimensional motion, bilateral coordination, and fine and rapid motor skills, still needs to be investigated.

The objective of our study was to quantify expert violinists’ motor adaptation to DAS provided as an anti-gravitational force by a passive device at the left elbow by assessing upper-limb joint kinematics, electromyography (EMG), and musical performance. Based on motor adaptation literature, quantifying adaptation requires the succession of the following conditions: (1) a null-field control condition to assess baseline performance levels; (2) a force-field (DAS in our study) adaptation condition to assess changes in performance, especially during early and late adaptation; and (3) a null-field (no DAS) washout condition to assess aftereffects. Quantifying savings requires repeating these three conditions at a later time. We hypothesized that violinists would be perturbed by DAS during early adaptation but would adapt to its anti-gravitational force upon repeated exposure. Specifically, we hypothesized that compared with control (null field), introducing DAS (force field) in early adaptation would increase joint angular root mean square errors (RMSE) and decrease similarity scores for joint angles and muscle activations, as well as musical performance scores. In line with motor adaptation literature (Scalona et al., 2018; Shadmehr & Mussa-Ivaldi, 1994), we also hypothesized that by late adaptation (force field), joint angular RMSE would decrease and that similarity scores for joint angles and muscle activations as well as musical performance scores would increase when violinists repeatedly played a single musical excerpt with DAS. Removing DAS following adaption during a washout (null field) condition should cause aftereffects. Aftereffects would translate into a sudden increase in RMSE and decrease in similarity scores for joint angles and muscle activations, and in musical performance scores, when compared with control values. As for muscle activation levels, we hypothesized that DAS would reduce left-side activity. Finally, we hypothesized that participants would be less perturbed by DAS when reexposed to its anti-gravitational force 1 week later.

## Method

### Participants

Fifteen (10 female) volunteer violinists (age: 31.3 ± 8.9 years; mass: 70.9 ± 16.3 kg; height: 1.7 ± .1 m; violin experience: 25.0 ± 9.1 years; mean ± SD) were recruited. Inclusion criteria included playing the violin as primary occupation. Five participants were professionals, two were semi-professionals, and eight were university music majors. Exclusion criteria included undergoing general anesthesia and having any pain (or diagnosis) in the upper limbs that would prevent musicians from playing at their usual level of performance within the past 12 months. This research complied with the American Psychological Association Code of Ethics and was approved by the University of Montreal’s research ethics committee (#18–005-P). Each participant provided informed consent.

### Instrumentation

#### Kinematics

**Figure 1:**
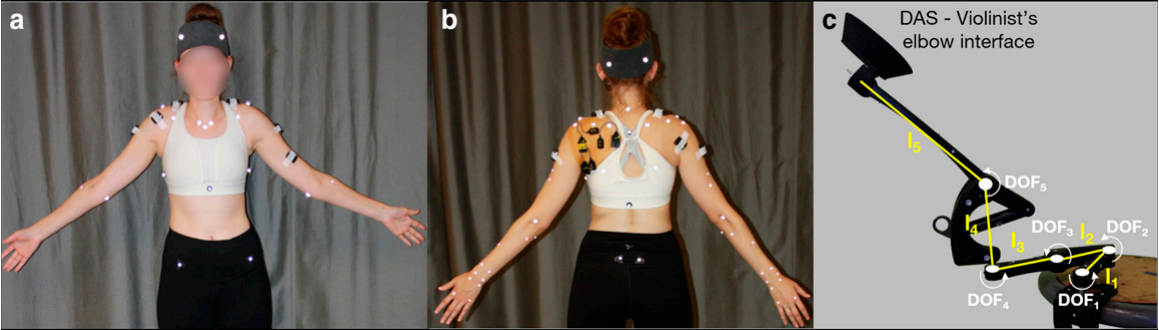
(a) Anterior and (b) posterior views of a participant equipped with reflective skin markers and EMG electrodes. (c) A picture of the passive device used for DAS with degrees of freedom (DOF_1-5_) represented. All DOFs have vertical axes of rotation except for DOF_5_’s, which is horizontal thus enabling DAS-elbow interface motion in the sagittal plane. Segments’ lengths are: l_1_ = 130 mm, l_2_ = 105 mm, l_3_ = 130 mm, l_4_ = 145 mm, and l_5_ = 250 mm. EMG = electromyography; DAS = dynamic assistive support.

Participants were equipped with 61 reflective skin markers, placed on the pelvis (*n* = 4), thorax (*n* = 7), head (*n* = 4), both clavicles (*n* = 2 × 2), scapulae (*n* = 5 × 2), arms (*n* = 4 × 2), forearms (*n* = 8 × 2), and hands (*n* = 4 × 2; Figure 1a–b). We followed the International Society of Biomechanics recommendations for marker placement (Wu et al., 2005) and incorporated modifications (Jackson et al., 2012; Michaud et al., 2016). An optoelectronic system of 18 cameras (VICON, Oxford, UK) tracked marker trajectories at a sampling frequency of 100 Hz.

#### Electromyography

After proper skin preparation, wireless surface EMG electrodes (Trigno Legacy sensors, dual on-board stabilizing reference, 26 × 37 x 15 mm body size, 10 mm inter-electrode spacing, Delsys, Natick, MA, USA) were placed following SENIAM recommendations (Hermens et al., 2000) on muscle bellies of the participant’s left sternocleidomastoid, upper trapezius, medial deltoid, biceps brachii, and right upper trapezius, anterior, medial, and posterior deltoids (Figure 1a–b). Under sterile conditions, we inserted intramuscular paired hook fine-wire electrodes (30 mm x 27 ga, Natus Neurology, Middleton, WI, USA) into the left infraspinatus, supraspinatus, and subscapularis (lower fibers), based on recommendations from Kadaba et al. (1992) and Morris et al. (1998). Due to poor scapular winging, we were only able to insert an electrode into the subscapularis of four participants and thus did not include this muscle in our analyses. Signal quality was verified by manual testing and visualization of online EMG recordings (Kadaba et al., 1992; Morris et al., 1998). Muscle activity was recorded at a sampling frequency of 2000 Hz.

#### Audio

Sound was recorded using a ZOOM H4n (ZOOM Corporation, Tokyo, Japan) recorder at a sampling frequency of 44100 Hz.

#### Dynamic assistive support (DAS)

Support was provided by a passive (i.e., without actuators) spring-based device that applies an upward vertical force at the elbow to counteract the effect of gravity at the upper limb and moves freely in all three planes (Kinova O110, Boisbriand, QC, Canada). The range of motion of the distal end of the DAS is larger than the one of the violinists’ elbows to avoid users’ joint motion restrictions. This device attaches to an external object (here a stool placed at hip level to the musician’s left) and thus does not add its weight onto the user like an exoskeleton. We designed and 3D printed a piece that replaces the original forearm brace to support the elbow without imposing a specific upper-limb configuration (Figure 1c). Assistance was adjusted to support ~80% of the participant’s arm and violin weights.

### Experimental Protocol

Participants visited the laboratory twice, interspersed with 6.1 ± 2.7 days. The first visit (*Week_1_*) was to quantify motor adaptation to DAS, whereas the second visit (*Week_2_*) was to assess potential savings. Preliminary measurements and experimental conditions described thereafter were identical for *Week_1_* and *Week_2_*.

### Preliminary Measurements

#### Static pose and functional movements

Once equipped, participants first held a standing static pose for a few seconds and then completed a series of functional movements to locate joint centers and axes of rotation (Jackson et al., 2012), and create a personalized multibody kinematic model. Functional movements involved trunk and neck flexion-extension, rotation and circumduction, elbow flexion-extension, radioulnar pronation-supination, and wrist flexion-extension, adduction-abduction and rotation.

*Maximal voluntary contractions (MVCs)*. Participants completed a set of seven 3-s MVCs (Dal Maso et al., 2016; Table 1) to get muscles’ maximal activations. MVCs were completed in a randomized order and repeated twice. Thirty-second breaks were given between two repetitions of the same MVC, and a 1-min break was given between different MVC.

**Table 1:** Description of MVC Tests

Side	Position	
Left and right	Participant is in a seated position with the arm abducted at 90°, neck bent to the ipsilateral side, head rotated to the contralateral side, and palm of hand facing down. Tester resists participant’s arm abduction by applying force at the head and elbow.	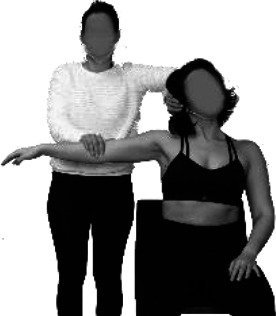
Left and right	Participant is in a seated position with the arm flexed at 90° and the palm of the hand facing down. Tester resists participant’s arm flexion by applying force at the elbow.	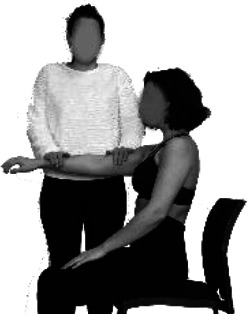
Left and right	Participant is in a ground-facing prone position with the arm horizontally flexed at 90° in line with the lower trapezius muscle fibers and the thumb pointing upward. Tester resists participant’s horizontal arm flexion by applying force at the elbow.	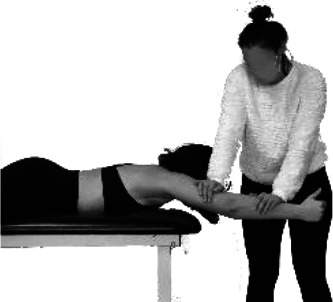
Right	Participant is in a ground-facing prone position with the arm horizontally abducted at 90° and the elbow flexed at 90°. Tester resists participant’s horizontal arm abduction by applying force at the elbow.	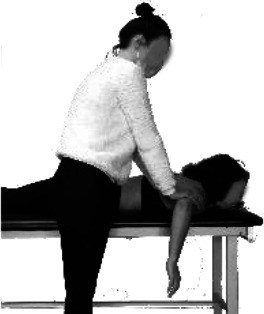

*Note*. MVC = maximal voluntary contraction.

### Experimental Conditions

**Figure 2:**
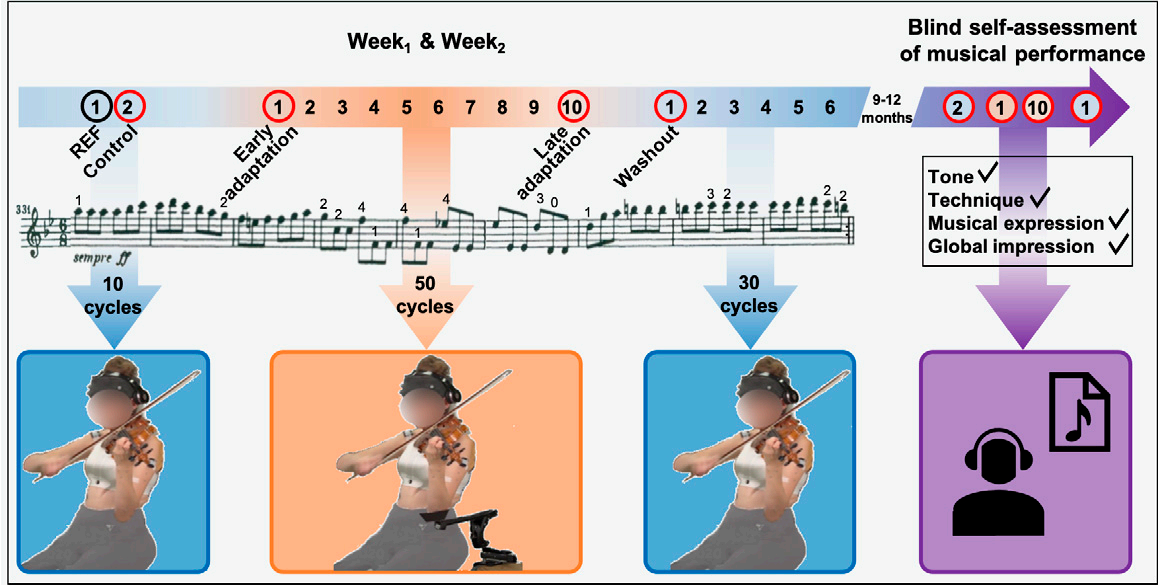
Experimental conditions completed during *Week_1_* and *Week_2_*. No DAS conditions (null field) are represented in blue, while the DAS condition (force field) is in orange. Arrows indicate how many cycles were played on a loop for each condition. For each cycle, the music score was played once and numbers above notes indicate left fingering. Each five consecutive cycles was averaged into a block of data. The black circle shows the block of data used as reference (*REF*), while red circles indicate blocks used for analysis, namely, *Control*, *Early adaptation*, *Late adaptation*, and *Washout*. The final experimental condition was completed 9–12 months after *Week_2_* and consisted in blindly assessing one’s own musical performance, recorded during *Control, Early adaptation*, *Late adaptation*, and *Washout* red-circled blocks (*Week_1_* and *Week_2_*)*,* on the following adjudication criteria: tone, technique, musical expression, and global impression. DAS = dynamic assistive support.

Violinists played a 13-s musical excerpt from Beethoven’s Symphony No. 9 (Movement IV) on a loop. They were asked to maintain dynamics and musical intentions throughout the experiment. We chose this excerpt because it is representative of the motor repertoire played by violinists in orchestra and include fast and varied left finger movements that require position shifting and to play on different strings. The excerpt was played at a tempo of 84 bpm provided to the participants via headphones (Beats Electronics, LLC, Santa Monica, CA, USA). We sent the music score with annotated fingering (Figure 2) to the participants 1 week prior to *Week_1_* to ensure identical left finger patterns between musicians. All practiced the piece before the day of the experiment and were able to play it accurately and comfortably. For both *Week_1_* and *Week_2_*, the succession of the experimental conditions (Figure 2) was as follows:

1. *Control*. Participants played the excerpt 10 times on a loop with no DAS.
2. *Adaptation*. Participants played the excerpt 50 times on a loop with DAS.
3. *Washout*. Participants played the excerpt 30 times on a loop with no DAS.

Two-minute breaks were given in between experimental conditions.

Finally, 9–12 months after *Week_2_*, participants completed a self-assessment of their musical performance (Figure 2). We sent them eight audio files of their own performance. Audio files consisted of five consecutive excerpts played during control, early and late adaptation, and early washout conditions of both *Week_1_* and *Week_2_*. Files were randomly numbered from one to eight to ensure blind assessment. For each audio file, participants had to attribute themselves a grade from 1 (worst) to 10 (best) on standard musical adjudication criteria, namely, tone, technique, musical expression, and global impression. They also had to indicate whether they thought the excerpt had been played using DAS, by answering “yes,” “no,” or “I don’t know.”

### Data Preprocessing

#### Joint kinematics

Centers of rotation of the trunk, wrist, and neck joints and axes of rotations of the elbow and radioulnar joints were determined using functional methods (Ehrig et al., 2006; O’Brien et al., 2000). An anatomical method was used to place sternoclavicular, acromioclavicular, and glenohumeral centers of rotations (Michaud et al., 2016). Joint kinematics were then reconstructed using a nonlinear least-squares algorithm (Thouzé et al., 2016). The multibody kinematic model included 15 segments, namely, pelvis, thorax, head, both clavicles, scapulae, arms, proximal and distal extremities of the forearms, and hands. Rigid body segments were actuated by 47 degrees of freedom, namely, three rotations and translations for the pelvis, thorax, and both arms; three rotations of the head, both clavicles, and scapulae; and two rotations for both forearms and hands. Head rotations were excluded as they presented large intra-participant variability. Due to corrupted data files, joint kinematics recorded during one participant’s *Week_1_* was not reconstructed.

#### EMG

All filters mentioned thereafter are zero-lag second-order Butterworth filters. Raw EMG signals were digitally band-pass filtered between 10 and 400 Hz. Visual inspection of the EMG power spectrum revealed 60 Hz electrical noise contamination. EMG signals were thus also notch filtered between 59 and 61 Hz. To obtain linear EMG envelops, signals were rectified and low-pass filtered at 9 Hz. To determine the maximal activation of each muscle, EMG envelopes of all MVC trials were concatenated and the median value of the 2000 (1 s) highest nonconsecutive data points was kept. EMG data were then normalized using corresponding maximal muscle activations to get activation levels. Due to electrode malfunctions, we had to exclude the biceps brachii from analyses. Finally, one participant’s *Week_1_* and another *Week_2_* EMG data were excluded due to technical difficulties with our acquisition system.

### Data Processing

Kinematic, EMG, and sound data were divided into cycles corresponding to one musical excerpt each. We averaged joint angles and normalized EMG into blocks of five consecutive cycles listed thereafter and graphically presented in Figure 2:

- *REF*, corresponding to the first block of the control condition.
- *Control*, corresponding to the last block of the control condition.
- *Early adaptation*, corresponding to the first block of the adaptation condition.
- *Late adaptation*, corresponding to the last block of the adaptation condition.
- *Washout*, corresponding to the first block of the washout condition.

#### Root mean square error

We computed angular error as the RMSE of the difference between joint angles of *REF* and the four subsequent blocks of interest.

#### Cosine similarity

Cosine similarity provides a measure of similarity between two non-zero vectors (Steele et al., 2019). Computing the cosine of the angle between two vectors results in values ranging from 0 to 1, corresponding to no (i.e., vectors are perpendicular) and perfect (i.e., vectors are aligned) similarity, respectively (Steele et al., 2019). Cosine similarity was computed between *REF* and the four subsequent blocks of interest, for both joint angle and EMG activation data.

### Statistics

To quantify expert violinists’ motor adaptation and savings to DAS, we performed linear mixed models on each variable of interest: RMSE, mean muscle activations, cosine similarity for both joint angles and EMG activations, and each musical criterion evaluated (tone, technique, musical expression, and global impression). For each variable, we measured effects of *Exposure* (*Week_1_* and *Week_2_*), *Block* (*Control*, *Early adaptation*, *Late adaptation*, and *Washout*), and their interaction. Statistical significance threshold was set at α = .05. Significant *Block* effects were followed by post-hoc tests with Bonferroni correction to adjust *p* values for multiple comparisons. We only reported differences between *Control* and *Early adaptation*, *Early adaptation* and *Late adaptation*, and *Control* and *Washout* blocks, as these comparisons are most relevant in investigating motor adaptation. Effect sizes were reported using Cohen (2013)’s *d* and interpreted as very small (*d* < .2), small (0.2 ≤ *d* < .5), medium (0.5 ≤ *d* < .8), large (0.8 ≤ *d* < 1.2), very large (1.2 ≤ *d* < 2.0), or huge (*d* = 2.0; Sawilowsky, 2009). Additionally, to determine if participants could discriminate between audio files recorded while they were playing with and without DAS during the blind self-assessment of musical performance, we compared numbers of correct and wrong answers to the question “Do you think that you were using the mobility assistive device for this recording?” in DAS *versus* no DAS conditions, using Fisher’s exact test. “I don’t know” answers were coded as wrong. Statistical analyses were performed using SAS 9.4 (SAS Institute Inc., Cary, NC, USA).

## Results

### Joint Kinematics

#### Root mean square error

**Figure 3:**
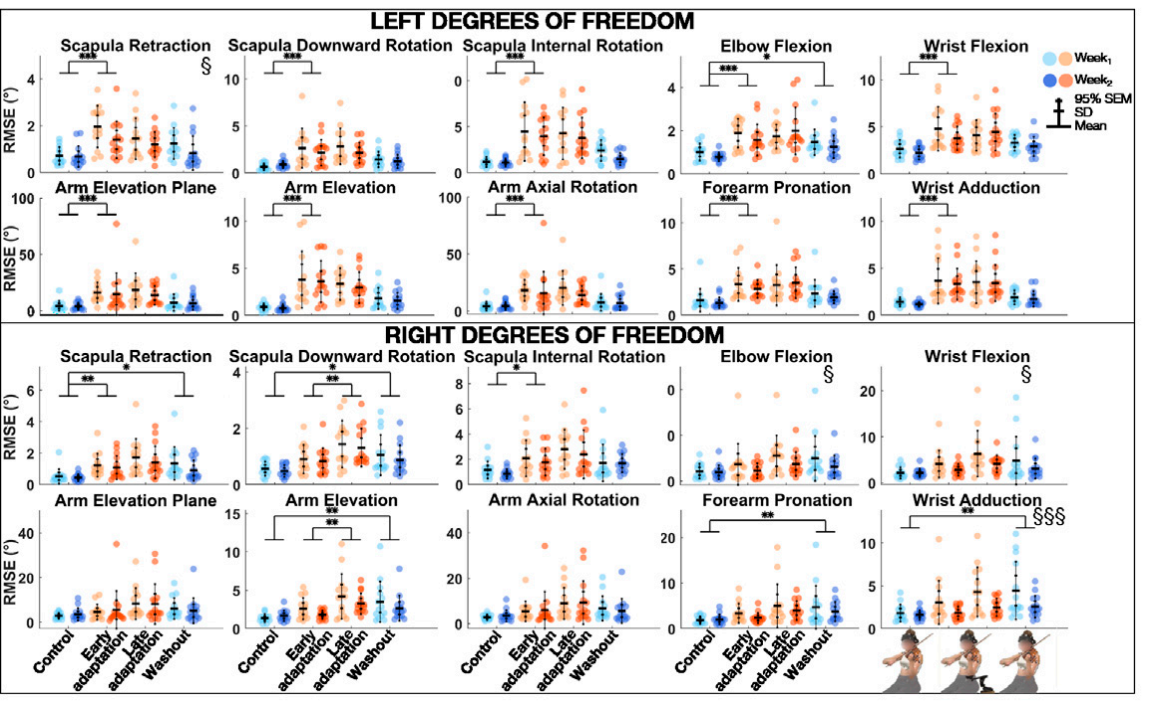
Group mean ± SD and 95% SEM and participants’ individual joint angular RMSE of left (top) and right (bottom) upper-limb degrees of freedom for *Control*, *Early adaptation*, *Late adaptation* and *Washout* blocks for *Week_1_* and *Week_2_.* Significant *Exposure* effects are shown with § (*p* ≤ .05) and §§§ (*p* ≤ .001), while significant differences between blocks are indicated with single, double, and triple asterisks for *p* ≤ .05, *p* ≤ .01, and *p* ≤ .001, respectively. RMSE = root mean square error.

The linear mixed model analysis (Table 2) revealed that RMSE was significantly smaller during *Week_2_* than *Week_1_* for left scapular retraction, and right elbow flexion, wrist adduction and flexion (Figure 3). *Block* effect was significant for all degrees of freedom. Post-hoc comparisons revealed that RMSE was significantly greater during *Early adaptation* than *Control* for all left degrees of freedom, as well as right scapular retraction and internal rotation, with large to very large effect sizes (0.86 ≤ *d* ≤ 1.40). During *Early adaptation*, violinists played with the left scapula less downwardly rotated but more externally rotated, the arm more elevated but less externally rotated, the elbow less flexed, the forearm more supinated, and the wrist more flexed and abducted. As for the right scapula, it was more protracted. In addition, RMSE was significantly greater during *Late adaptation* than *Early adaptation* for right scapular downward rotation and arm elevation, with medium to large effect sizes (0.72 ≤ *d* ≤ .87). Finally, RMSE was significantly higher during *Washout* than *Control* for left elbow flexion, as well as right scapular retraction and downward rotation, arm elevation, forearm pronation, and wrist adduction, with medium to large effect sizes (0.69 ≤ *d* ≤ .97).

**Table 2:** *p* Values of EXP*Block Linear Mixed Model Analyses of RMSE and Cosine Similarity of Joint Angles for All Degrees of Freedom

	Degrees of Freedom	RMSE						Cosine Similarity					
		Linear Mixed Model			Post-Hoc Block Comparisons			Linear Mixed Model			Post-Hoc Block Comparisons		
		EXP*Block Interaction	EXP	Block	Control VS Early adaptation	Early VS Late adaptation	Control VS Washout	EXP*Block Interaction	EXP	Block	Control VS Early adaptation	Early VS Late adaptation	Control VS Washout
Left Side	Scapular retraction	0.5015	**0.0158**	**<.0001**	**<.0001 (0.97)**	0.3207 (0.31)	0.3723 (0.55)	0.2576	0.7711	0.2025			
	Scapular downward rotation	0.5250	0.2150	**<.0001**	**<.0001 (0.92)**	1.0000 (0.07)	0.5025 (0.72)	0.6127	0.4693	0.1071			
	Scapular internal rotation	0.8599	0.1546	**<.0001**	**<.0001 (1.19)**	0.6183 (0.09)	1.0000 (0.77)	0.7682	0.3301	0.1595			
	Arm axial rotation	0.6285	0.2372	**<.0001**	**<.0001 (0.94)**	1.0000 (<.01)	1.0000 (0.79)	0.6947	0.7230	0.3852			
	Arm elevation plan	0.8457	0.3708	**<.0001**	**0.0004 (0.86)**	1.0000 (0.03)	1.0000 (0.79)	0.9734	0.6645	**0.0261**	0.3433 (0.67)	1.0000 (0.23)	1.0000 (0.48)
	Arm elevation	0.9894	0.4384	**<.0001**	**<.0001 (1.13)**	1.0000 (0.25)	0.2909 (0.86)	0.8380	0.1564	**0.0291**	0.0953 (0.69)	1.0000 (<.01)	1.0000 (0.53)
	Elbow flexion	0.3248	0.3041	**<.0001**	**<.0001 (1.29)**	1.0000 (0.14)	**0.0461 (0.90)**	0.5621	0.5400	**0.0175**	0.7001 (0.83)	0.6489 (0.24)	1.0000 (0.84)
	Forearm pronation	0.7407	0.3655	**<.0001**	**0.0002 (1.40)**	1.0000 (0.19)	0.4151 (0.93)	0.9865	**0.0485**	0.1296			
	Wrist adduction	0.9914	0.4561	**<.0001**	**<.0001 (1.02)**	1.0000 (<.01)	1.0000 (0.68)	0.8886	0.3698	**0.0012**	**0.0076 (0.53)**	1.0000 (0.09)	1.0000 (0.54)
	Wrist flexion	0.3484	0.1634	**<.0001**	**<.0001 (1.03)**	1.0000 (<.01)	0.4976 (0.64)	0.4804	0.3239	**0.0118**	**0.0340 (0.72)**	1.0000 (0.05)	1.0000 (0.42)
Right Side	Scapular retraction	0.8179	0.1061	**<.0001**	**0.0141 (1.29)**	0.3376 (0.68)	**0.0217 (0.97)**	0.4744	0.0747	0.0820			
	Scapular downward rotation	0.9874	0.2630	**<.0001**	0.1246 (0.71)	**0.0048 (0.72)**	**0.0198 (0.75)**	0.8237	0.7221	0.0971			
	Scapular internal rotation	0.9408	0.2622	**<.0001**	**0.0339 (0.98)**	0.2653 (0.40)	0.1946 (0.77)	0.9337	0.3703	0.2218			
	Arm axial rotation	0.9402	0.9168	**0.0042**	0.6882 (0.49)	0.2122 (0.56)	0.3601 (0.72)	0.9460	0.3612	0.0933			
	Arm elevation plan	0.9291	0.8829	**0.0171**	1.0000 (0.37)	0.2699 (0.46)	0.7064 (0.61)	0.8743	0.5129	0.2366			
	Arm elevation	0.4446	0.0730	**<.0001**	0.7407 (0.57)	**0.0048 (0.87)**	**0.0036 (0.75)**	0.4794	0.5331	**0.0078**	1.0000 (0.40)	0.0964 (0.44)	0.3372 (0.84)
	Elbow flexion	0.7403	**0.0282**	**0.0144**	1.0000 (0.35)	0.3037 (0.89)	0.1247 (0.69)	0.8399	0.0918	0.5603			
	Forearm pronation	0.8199	0.1643	**0.0015**	1.0000 (0.47)	0.1515 (0.63)	**0.0127 (0.69)**	0.7580	0.6177	0.6442			
	Wrist adduction	0.3320	**0.0005**	**0.0014**	0.9237 (0.47)	0.3982 (0.81)	**0.0037 (0.80)**	0.9868	0.7901	0.1832			
	Wrist flexion	0.5268	**0.0202**	**0.0019**	0.5967 (0.70)	0.1526 (0.65)	0.1349 (0.49)	0.3836	0.0791	0.1957			

*Note*. Significant *p* values (Cohen’s *d*) are shown in bold (*p* ≤ .05). RMSE = root mean square error.

### Cosine Similarity

**Figure 4:**
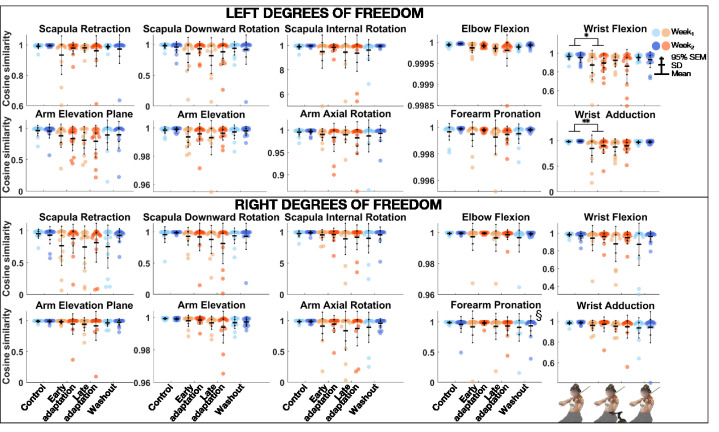
Group mean ± SD and 95% SEM and participants’ individual values of cosine similarity of joint angles of left (top) and right (bottom) upper-limb degrees of freedom between *REF* and *Control*, *Early adaptation*, *Late adaptation* and *Washout* blocks for *Week_1_* and *Week_2_.* Significant *Exposure* effect is shown with § (*p* ≤ .05), while significant differences between blocks are indicated with single and double asterisks for *p* ≤ .05 and *p* ≤ .01, respectively. REF = reference.

The linear mixed model analysis (Table 2) revealed that cosine similarity of joint angles between the *REF* block and *Control*, *Early adaptation*, *Late adaptation*, and *Washout* blocks was significantly lower during *Week_1_* than *Week_2_* for left forearm pronation (Figure 4). *Block* effect was significant for left arm elevation and elevation plan, elbow flexion, wrist adduction and flexion, and right arm elevation. Post-hoc comparisons revealed that cosine similarity between *REF* and *Control*, *Early adaptation*, *Late adaptation*, and *Washout* blocks was significantly lower during *Early adaptation* than *Control* for left wrist adduction and flexion, with medium effect sizes (0.53 ≤ *d* ≤ .72).

### Electromyography

#### Mean activation levels

**Figure 5:**
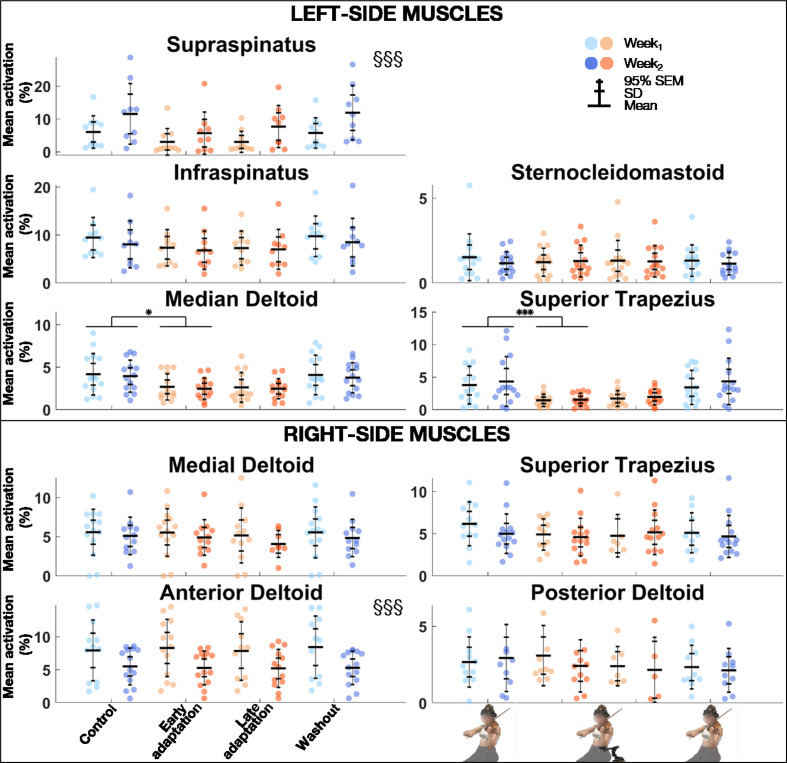
Group mean ± SD and 95% SEM and participants’ individual values of EMG activation levels of left (top) and right (bottom) muscles for *Control*, *Early adaptation*, *Late adaptation* and *Washout* blocks for *Week_1_* and *Week_2_.* Significant *Exposure* effects are shown with §§§ (*p* ≤ .001), while significant block differences are indicated with single and triple asterisks for *p* ≤ .05 and *p* ≤ .001, respectively. EMG = electromyography.

The linear mixed model analysis (Table 3) revealed that muscle activation levels were significantly greater for the left supraspinatus but smaller for the right anterior deltoid during *Week_2_* than *Week_1_* (Figure 5). *Block* effect was significant for the left supraspinatus, medial deltoid, and superior trapezius. Post-hoc comparisons revealed that muscle activation levels were significantly smaller during *Early adaptation* than *Control* for the left medial deltoid and superior trapezius, with large effect sizes (0.93 ≤ *d* ≤ 1.13).

**Table 3:** *p* Values of EXP*Block Linear Mixed Model Analyses of Mean Activations and Cosine Similarity of EMG Activations for All Muscles

	Muscles	Mean Activations						Cosine Similarity					
		Linear Mixed Model			Post-Hoc Block Comparisons			Linear Mixed Model			Post-Hoc Block Comparisons		
		EXP*Block Interaction	EXP	Block	Control VS Early adaptation	Early VS Late adaptation	Control VS Washout	EXP*Block Interaction	EXP	Block	Control VS Early adaptation	Early VS Late adaptation	Control VS Washout
Left Side	Supraspinatus	0.8276	**0.0012**	**0.0503**	0.1716 (1.03)	1.0000 (0.41)	1.0000 (0.01)	0.9456	0.7105	0.1438			
	Infraspinatus	0.9670	0.3662	0.2931				0.7532	**0.0116**	**0.0359**	0.2688 (0.80)	1.0000 (0.62)	0.3815 (0.59)
	Sternocleidomastoid	0.8621	0.4935	0.9777				0.2939	0.2939	0.4724			
	Superior trapezius	0.9237	0.3281	**<.0001**	**0.0008 (0.93)**	1.0000 (0.73)	1.0000 (0.23)	0.8922	0.2550	0.0762			
	Medial deltoid	0.9986	0.4949	**0.0011**	**0.0177 (1.13)**	1.0000 (0.08)	1.0000 (0.29)	0.9925	0.1743	**<.0001**	**0.0001 (1.25)**	1.0000 (0.28)	0.5353 (0.78)
Right Side	Superior trapezius	0.7655	0.4634	0.6174				0.7756	0.5412	**0.0241**	0.3115 (0.50)	1.0000 (0.50)	0.0779 (0.75)
	Anterior deltoid	0.9848	**0.0002**	0.9897				0.9506	0.1959	0.0985			
	Medial deltoid	0.9870	0.2099	0.8434				0.7314	0.2490	0.0595			
	Posterior deltoid	0.8568	0.6082	0.6429				0.9247	0.0898	0.1597			

*Note*. Significant *p* values (Cohen’s *d*) are shown in bold (*p* ≤ .05).

### Cosine Similarity

**Figure 6:**
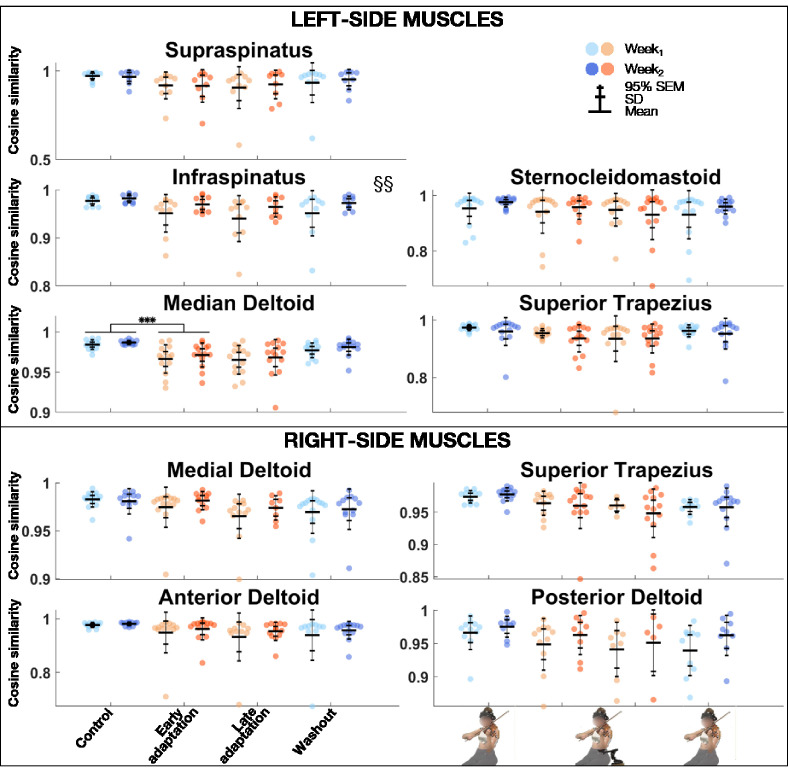
Group mean ± SD and 95% SEM and participants’ individual values of cosine similarity of EMG activations of left (top) and right (bottom) muscles between *REF* and *Control*, *Early adaptation*, *Late adaptation* and *Washout* blocks for *Week_1_* and *Week_2_.* Significant *Exposure* effect is shown with §§ (*p* ≤ .01), while significant block differences are indicated with triple asterisks for *p* ≤ .001. EMG = electromyography; REF = reference.

The linear mixed model analysis (Table 3) revealed that cosine similarity of EMG activations between the *REF* block and *Control*, *Early adaptation*, *Late adaptation*, and *Washout* blocks was significantly lower during *Week_1_* than *Week_2_* for left infraspinatus (Figure 6). *Block* effect was significant for the left infraspinatus, medial deltoid, and right superior trapezius. Post-hoc comparisons revealed that cosine similarity between the *REF* block and *Control*, *Early adaptation*, *Late adaptation*, and *Washout* blocks was significantly lower during *Early adaptation* than *Control* for the left medial deltoid, with a very large effect size (*d* = 1.25).

### Musical Performance

**Figure 7:**
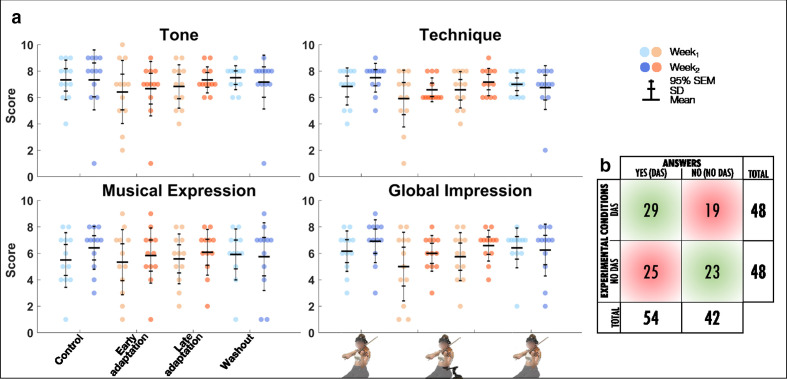
(a) Group mean ± SD and 95% SEM and participants’ individual self-assessed musical performance scores (/10) for tone, technique, musical expression, and global impression for *Control*, *Early adaptation*, *Late adaptation,* and *Washout* blocks for *Week_1_* and *Week_2_*. (b) Count of participants’ answers to the question “do you think that you were using the mobility assistive device for this recording?” for DAS and no DAS conditions. Correct and wrong answers are displayed in light green and red, respectively.

Twelve out of the 15 participants completed the self-assessment of musical performance. The linear mixed model analysis revealed that there were no effects of *Exposure* or *Block* for tone, technique, musical expression, or global impression (Table 4, Figure 7a). Fisher’s exact test revealed that frequencies of correct (52/96) and wrong (44/96) answers to the question “Do you think that you were using the mobility assistive device for this recording?” were not significantly different (*p* = .3058) with a sensitivity and specificity of 60% and 48%, respectively (Figure 7b).

**Table 4:** *p* Values of EXP*Block Linear Mixed Model Analyses of Self-Attributed Musical Performance Scores

Adjudication Criteria	Scores		
	Linear Mixed Model		
	EXP*Block Interaction	EXP	Block
Tone	0.8724	0.7778	0.3837
Technique	0.5939	0.1400	0.1342
Musical expression	0.8379	0.3021	0.9363
Global impression	0.6536	0.0928	0.1937

## Discussion

To the best of our knowledge, this study is the first to assess motor adaptation to a mobility assistive device for a motor task as highly specialized as violin playing. Our results revealed that DAS perturbed user kinematics but that cosine similarity of joint angles and EMG activations remained very strong. EMG activity levels were either similar or reduced with DAS. Finally, analysis of the blind self-assessment of musical performance revealed that violinists rated their performances similarly with and without DAS and were unable to discriminate between playing with and without the device. These findings shed light on the possible use of DAS in a musical and occupational setting, and are encouraging for the investigation of its role in limiting muscle fatigue accumulation in professional violinists.

### Motor Adaptation to DAS

Performing a task in a new dynamic environment leads to a mismatch between predicted sensory feedback and actual feedback (Ito, 2000; Shadmehr & Mussa-Ivaldi, 1994; Synofzik et al., 2006). This mismatch typically perturbs participants’ movements and leads to performance errors (Ito, 2000; Shadmehr & Mussa-Ivaldi, 1994). Here, introducing DAS increased RMSE during *Early adaptation* indicating that participants’ movements were perturbed. This perturbation not only affected all left-side degrees of freedom (scapula, arm, forearm, and hand) where DAS was applied but also the right scapula. Considering that no assistance was provided on that side, perturbation observed at the right scapula might be resulting from changes in left upper-limb kinematics, as both limbs coordinate to play and form a closed kinematic loop via the bow-violin interface.

To minimize prediction errors caused by motor perturbations, participants must update their internal models via a process called adaptation (Shadmehr & Mussa-Ivaldi, 1994). This phenomenon takes place upon repeated exposure to the perturbation (Imamizu et al., 2000; Scalona et al., 2018; Shadmehr & Mussa-Ivaldi, 1994; Synofzik et al., 2006), and thus we had hypothesized that violinists would adapt to DAS after repeating the excerpt 50 times. Despite our expectations, post-hoc comparisons between *Early* and *Late adaptation* revealed that RMSE of the left upper limb did not significantly decrease throughout adaptation. Absence of motor adaptation is further highlighted by increased RMSE during adaptation for scapula downward rotation and arm elevation, which were not perturbed in *Early adaptation*. A crucial element for updating internal models is the detection of errors (Ito, 2000; Shadmehr & Mussa-Ivaldi, 1994; Synofzik et al., 2006). In our study, participants received both proprioceptive and auditory feedbacks of their musical performances. Violinists were however instructed to play the excerpt as accurately as possible from a musical standpoint, and no instruction was given regarding motor patterns. To achieve this goal (i.e., play the excerpt correctly), violinists had to rely on auditory feedback. Results however showed no *Block* or *Exposure* effects on the musical criteria evaluated, and Fisher’s exact test revealed that violinists were unable to discriminate between playing with or without DAS. Therefore, we suggest that the absence of RMSE reduction during the adaptation period reflects the lack of predicted auditory feedback errors required to drive motor adaptation.

During washout (DAS removed), RMSE increased significantly for left elbow flexion, right scapula retraction and downward rotation, arm elevation, forearm pronation, and wrist adduction, compared with *Control*. Considering that no motor adaptation was witnessed for these degrees of freedom, we could not associate this error increase with aftereffects, which would have been a carry-over effect of the novel internal model constructed during motor adaptation to DAS (Shadmehr & Mussa-Ivaldi, 1994).

Although no motor adaptation to DAS was observed during the adaptation period, results showed significant RMSE decrease during *Week_2_* for the left scapula retraction, right elbow flexion, and wrist flexion and adduction. Since sensorimotor learning was not observed when comparing *Early* and *Late adaptation*, savings are unlikely. Significant *Exposure* effect might instead be a sign of habituation due to previous exposure to DAS, so that DAS-resulting posture during *Week_2_* was closer to the *REF* than *Week_1_*. Nonetheless, our results suggest that expert violinists can maintain their musical performances despite changing instrument setups, that is, DAS *versus* no DAS, indicating that professional musicians could alternate between rehearsing with assistance to playing a concert assistance-free without detrimental effects to their performances.

Maintenance of musical performance despite different instrumental setups had already been observed with the introduction of shoulder rests—which elevate the violin (Rabuffetti et al., 2007). Indeed, Rabuffetti et al. (2007) found that expert violinists could play scales with no and different heights of shoulder rests, without affecting sound quality (Rabuffetti et al., 2007). Increasing shoulder-rest height significantly changed left shoulder flexion and internal rotation, elbow flexion, forearm pronation, and wrist ulnar rotation, without perturbing right-side kinematics (Rabuffetti et al., 2007). In agreement with their results, we state that expert violinists’ sound production techniques were preserved despite upper-limb kinematic changes induced by DAS. Ability to sustain musical performance may come from the intrinsic motor variability displayed by experts completing a repetitive task, as is the case in our study (Bartlett et al., 2007; Srinivasan & Mathiassen, 2012). Motor variability is often displayed by experts and can serve several purposes (Bartlett et al., 2007; Konczak et al., 2009; Srinivasan & Mathiassen, 2012). One can be not to overload musculoskeletal structures during repetitive motion and thus is thought to protect from injuries (Bartlett et al., 2007; Srinivasan & Mathiassen, 2012). Another, which is specific to music, allows musicians to express a single note in a myriad of ways, conveying different emotions (Konczak et al., 2009). In our study, cosine similarity analyses revealed that violinists overall motor and EMG activation patterns remained similar across the different conditions. Indeed, similarity between *REF* and *Control*, *Early adaptation*, *Late adaptation*, and *Washout* blocks remained strong for both joint angles and EMG activations. Post-hoc comparisons of joint angles did show a weaker similarity during *Early adaptation* than *Control* for left wrist flexion and adduction, but these differences were driven by a few participants only (mean range: 0.85–0.90). As for post-hoc comparisons of EMG activations, they revealed that cosine similarity was weaker during *Early adaptation* than *Control* for the left medial deltoid, though it remained strong (>.95). As cosine similarity was strong across conditions for all degrees of freedom and muscles, we suggest that its values reflect subtle motor variability. Additionally, DAS decreased left external rotation, which has been linked to playing-related musculoskeletal disorders (Mizrahi, 2020). DAS-resulting limb configuration thus might be helpful in preventing injuries.

### Reduced Muscle Activations With DAS

Passive upper-extremity exoskeletons and mobility assistive devices have been shown to reduce muscle activations of the deltoid and/or trapezius during occupational tasks (Hall & Crouch, 2020; Huysamen et al., 2018; Iranzo et al., 2020; Kim et al., 2018; Maurice et al., 2020, Schmalz et al., 2019; Van Engelhoven et al., 2019). Our results extend this finding to violin playing, which involves tridimensional motion, bilateral coordination, and fine and rapid motor skills, and adds to it by showing that reduced activations with assistance can remain low overtime. Post-hoc comparisons between *Early* and *Late adaptation* were indeed not significant, indicating that left medial deltoid and superior trapezius activations remained similar throughout the adaptation condition, which lasted almost 11 min. Interestingly, decreased medial deltoid and upper trapezius activations occurred while violinists had their left scapulae less downwardly rotated and left arms more elevated. Without DAS, these joint actions would have required greater muscle activations (McKinley et al., 2015). As this was not the case, we can suggest that reduced muscle activations were due directly to DAS support. Similar results were observed for the supraspinatus though *Block* effect was only marginally significant and post-hoc comparisons were inconclusive. Although our results are not sufficient to draw conclusions on the possible effects of DAS on muscle fatigue, the decrease in some muscles’ activities is promising. Future studies should investigate effect of passive assistance on muscle fatigue, as it may have implications for playing-related injury prevention.

### Limitations

The duration of the selected musical excerpt, which reduced the possible number of times played per condition, constitutes a limit of our work. Indeed, tasks used in motor adaptation studies typically last much less than 13 s, and more trials are completed during the adaptation period, from 80 up to 1000, *versus* 50 in our study (Izawa & Shadmehr, 2011; Kitago et al., 2013; Pekny et al., 2011; Scalona et al., 2018; Shadmehr & Mussa-Ivaldi, 1994), although we can consider that participants had more time to adapt throughout each trial. Huberdeau et al. (2015) also showed that 10 trials could be enough to elicit motor adaptation. Moreover, proper assessment of savings would have required that participants do not play their instruments in between *Week_1_* and *Week_2_*, as performing the evaluated task without perturbation in-between experiments could prevent formation of savings (Kitago et al., 2013). However, it would have been impossible to recruit professional violinists under such experimental constraints. Finally, only 12 out of the 15 participants completed the blind self-assessment of musical performance, nonetheless representing a high retention rate (80%) considering that this experimental condition was completed 9–12 months after laboratory testing.

### Conclusion

We had hypothesized that violinists would adapt to the motor perturbation caused by DAS. Considering that RMSE did not decrease throughout adaptation however, we conclude that violinists did not adapt to DAS. Indeed, musicians lacked the predicted auditory feedback errors required to adjust their internal models and adapt to DAS. Nonetheless, joint angles and EMG activity were strongly similar with and without assistance, indicating that violinists’ original motor patterns remained with DAS. Furthermore, expert violinists were unable to differentiate audio excerpts played with and without DAS, indicating that musical performance was maintained with DAS. Finally, the decrease in mean activations of the left anterior deltoid and superior trapezius indicates that DAS may limit muscle fatigue accumulation and might prevent playing-related injuries.

## Key Points

- Motor adaptation of expert violinists to DAS applied to the left elbow (violin-holding side) was investigated while participants played their instruments.
- Violinists’ left kinematics were perturbed by DAS, but no motor adaptation was observed due to similar musical performance with and without assistance.
- Muscle activations of the medial deltoid and superior trapezius of the assisted side were reduced with DAS. Effect of DAS on muscle fatigue should be investigated in future studies.
- Violinists’ motor patterns remained similar with and without assistance.
